# Association between SARS-CoV-2 RNAemia, skewed T cell responses, inflammation, and severity in hospitalized COVID-19 people living with HIV

**DOI:** 10.1016/j.isci.2023.108673

**Published:** 2023-12-07

**Authors:** Matteo Augello, Valeria Bono, Roberta Rovito, Camilla Tincati, Silvia Bianchi, Lucia Taramasso, Antonio Di Biagio, Annapaola Callegaro, Franco Maggiolo, Elisa Borghi, Antonella d’Arminio Monforte, Giulia Marchetti

**Affiliations:** 1 Clinic of Infectious Diseases and Tropical Medicine, San Paolo Hospital, ASST Santi Paolo e Carlo, Department of Health Sciences, University of Milan, Milan, Italy; 2 Microbiology and Clinical Microbiology, Department of Health Sciences, University of Milan, Milan, Italy; 3 Infectious Diseases Unit, San Martino Policlinico Hospital, Genoa, Italy; 4 Department of Health Sciences, University of Genoa, Genoa, Italy; 5 Biobank Unit and Microbiology and Virology Laboratory, ASST Papa Giovanni XXIII, Bergamo, Italy; 6 Division of Infectious Diseases, ASST Papa Giovanni XXIII, Bergamo, Italy

**Keywords:** biological sciences, Immunology, immune response, Microbiology, Virology

## Abstract

Severe COVID-19 outcomes have been reported in people living with HIV (PLWH), yet the underlying pathogenetic factors are largely unknown. We therefore aimed to assess SARS-CoV-2 RNAemia and plasma cytokines in PLWH hospitalized for COVID-19 pneumonia, exploring associations with magnitude and functionality of SARS-CoV-2-specific immune responses.

Eighteen unvaccinated PLWH (16/18 on cART; median CD4 T cell count 361.5/μL; HIV-RNA<50 cp/mL in 15/18) and 18 age/sex-matched people without HIV were consecutively recruited at a median time of 10 days from symptoms onset. PLWH showed greater SARS-CoV-2 RNAemia, a distinct plasma cytokine profile, and worse respiratory function (lower P_a_O_2_/F_i_O_2_*nadir*), all correlating with skewed T cell responses (higher perforin production by cytotoxic T cells as well as fewer and less polyfunctional SARS-CoV-2-specific T cells), despite preserved humoral immunity.

In conclusion, these data suggest a link between HIV-related T cell dysfunction and poor control over SARS-CoV-2 replication/dissemination that may in turn influence COVID-19 severity in PLWH.

## Highlights

• PLWH hospitalized for COVID-19 pneumonia feature higher SARS-CoV-2 RNAemia
• PLWH are also characterized by a distinct plasma cytokine pattern
• Unlike humoral immunity, SARS-CoV-2-specific T cell responses are skewed in PLWH
• Skewed T cell responses associate with higher RNAemia, inflammation, and severity

## Graphical abstract

**Figure:**
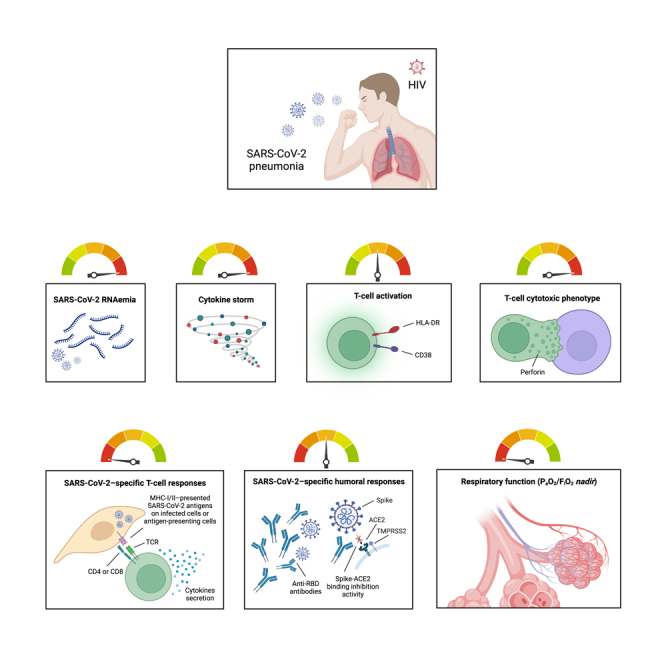

## Introduction

People living with HIV (PLWH) may display an increased risk of severe COVID-19 outcomes,^1^^,^^2^^,^^3^^,^^4^^,^^5^^,^^6^^,^^7^^,^^8^^,^^9^^,^^10^^,^^11^^,^^12^^,^^13^ yet the underlying pathogenetic factors have not been fully characterized.

The pathogenesis of severe COVID-19 in the general population has been associated to both host factors, among which altered immune responses and exacerbated inflammation, as well as viral factors, such as SARS-CoV-2 dissemination outside the respiratory tract into the bloodstream.^14^

While neutralizing antibodies protect from SARS-CoV-2 infection, a prompt and coordinated development of both T-cellular and humoral immunity is required to control viral replication and to protect from severe disease.^15^^,^^16^^,^^17^^,^^18^^,^^19^^,^^20^^,^^21^^,^^22^ Since HIV infection is characterized by a profound disruption of the innate and adaptive immune system,^23^^,^^24^^,^^25^ concerns regarding the ability of PLWH to mount an adequate response to SARS-CoV-2 have arisen since the beginning of the pandemic. Nonetheless, studies assessing SARS-CoV-2-specific adaptive immunity in PLWH are limited and yielded inconsistent findings,^2^^,^^26^^,^^27^^,^^28^^,^^29^^,^^30^^,^^31^^,^^32^^,^^33^^,^^34^^,^^35^^,^^36^^,^^37^^,^^38^^,^^39^^,^^40^ yet overall suggesting that it may be less efficient than in the general population, especially in individuals with low CD4 T cell counts and uncontrolled HIV viremia. However, such studies mainly focused on PLWH recovered from mild COVID-19,^26^^,^^27^^,^^28^^,^^29^^,^^30^^,^^31^^,^^32^^,^^33^^,^^34^^,^^39^ with only few evaluating immune responses during the acute phase of severe disease.^35^^,^^36^^,^^37^^,^^38^ Moreover, the majority of studies focused on humoral immunity, while SARS-CoV-2-specific T cell responses have been less consistently investigated in PLWH.^26^^,^^27^^,^^33^^,^^34^^,^^35^^,^^36^

Elevated plasma concentrations of proinflammatory cytokines have been shown to associate with a higher clinical severity and mortality in individuals with COVID-19.^41^^,^^42^^,^^43^^,^^44^^,^^45^^,^^46^^,^^47^ Given that HIV infection elicits a chronic hyperinflammatory state,^48^^,^^49^ it may be speculated that such HIV-driven inflammation may further worsen COVID-19-related cytokine storm. However, an opposite model could also be speculated, in which the HIV-driven immune deficit may shelter PLWH from COVID-19-related immunopathology. Nevertheless, data concerning the cytokine storm during COVID-19 in PLWH are scarce and conflicting.^2^^,^^33^^,^^34^^,^^50^

Poor control of SARS-CoV-2 replication/dissemination, as estimated through SARS-CoV-2 RNAemia,^51^^,^^52^^,^^53^ has been associated with blunted SARS-CoV-2-specific immune responses,^51^^,^^54^^,^^55^^,^^56^ heightened inflammation,^52^^,^^57^ higher markers of tissue damage,^58^ as well as worse clinical severity and outcomes.^52^^,^^57^^,^^58^^,^^59^^,^^60^^,^^61^^,^^62^^,^^63^ Several cases of prolonged SARS-CoV-2 infection with delayed viral clearance in the upper respiratory tract has been reported in PLWH with severe T cell depletion,^64^^,^^65^^,^^66^^,^^67^ suggesting that T cell dysfunction may hamper viral control. Nonetheless, the role of SARS-CoV-2 RNAemia in the pathogenesis of COVID-19 in PLWH has not been determined.

We hereby assessed SARS-CoV-2 RNAemia and the plasma cytokine *milieu* in a cohort of unvaccinated PLWH hospitalized for acute COVID-19 pneumonia as compared to HIV-negative individuals, and explored their association with the magnitude and functionality of SARS-CoV-2-specific T cell and humoral responses.

## Results

### Study design and population

In this cross-sectional study, 18 SARS-CoV-2-unvaccinated PLWH and 18 age- and sex-matched HIV-negative individuals hospitalized for acute COVID-19 pneumonia were consecutively enrolled between March 2020 and January 2021 at one of the participating Infectious Diseases centers in Northern Italy, at a median time of 10 (IQR: 6.75–13) days after symptoms onset.

Participants characteristics are reported in Table 1. The two study groups were comparable in terms of age [60 (IQR: 48–67) years], sex, ethnicity, and comorbidities. PLWH had a lower arterial partial pressure of oxygen (P_a_O_2_)/fraction of inspired oxygen (F_i_O_2_) *nadir* (*p* = 0.0005) compared to HIV-negative individuals. One in-hospital death (5.6%) was registered among PLWH compared to no one in the HIV-negative control group, yet the difference was not statistically significant.

**Table 1:** Demographic and clinical characteristics of study participants

	All study population (*n* = 36)	HIV+ (*n* = 18)	HIV– (*n* = 18)	*p* value HIV+ vs*.* HIV–**∗**
**Age, years** [median (IQR)]	60 (48–67)	60 (49–67)	60 (47–66)	0.7843
**Sex** [*n* (%)]				>0.9999
Male	28 (77.8)	14 (77.8)	14 (77.8)	
Female	8 (22.2)	4 (22.2)	4 (22.2)	
**Ethnicity** [*n* (%)]				0.0903
Caucasian	28 (77.8)	15 (83.3)	13 (72.2)	
Latin-American	6 (16.7)	1 (5.6)	5 (27.8)	
African	2 (5.5)	2 (11.1)	0 (0)	
**Comorbidities** [*n* (%)]				
Hypertension	14 (38.9)	9 (50)	5 (27.8)	0.3053
Chronic heart disease	9 (25)	4 (22.2)	5 (27.8)	>0.9999
Chronic pulmonary disease	2 (5.5)	1 (5.6)	1 (5.6)	>0.9999
Chronic kidney disease	4 (11.1)	3 (75)	1 (25)	0.6026
Liver disease	6 (16.7)	5 (27.8)	1 (5.6)	0.1774
Diabetes	8 (22.2)	2 (11.1)	6 (33.3)	0.2285
Neurologic disease	5 (13.9)	4 (22.2)	1 (5.6)	0.3377
History of cancer	7 (19.4)	5 (27.8)	2 (11.1)	0.4018
**Symptoms at hospital admission** [*n* (%)]				
Fever	32 (88.9)	15 (83.3)	17 (94.4)	0.6026
Fatigue	15 (41.7)	7 (38.9)	8 (44.4)	>0.9999
Arthromyalgia	5 (13.9)	3 (16.7)	2 (11.1)	>0.9999
Cough	18 (50)	6 (33.3)	12 (66.7)	0.0943
Dyspnea	25 (69.4)	11 (61.1)	14 (77.8)	0.4705
Diarrhea	12 (33.3)	4 (22.2)	8 (44.4)	0.2890
Anosmia/dysgeusia	8 (22.2)	5 (27.8)	3 (16.7)	0.6906
**Duration of symptoms before biological samples collection, days** [median (IQR)]	10 (6.75–13)	9.5 (6.5–12.5)	10 (6.75–13)	0.6983
**P**_**a**_**O**_**2**_**/F**_**i**_**O**_**2**_***nadir*** [median (IQR)]	167 (146–248)	140 (122–151.5)	207 (156.3–309.3)	**0.0005**
**Blood exams upon admission** [median (IQR)]				
Leukocytes, 10^3^/μL	6.01 (4.38–8.87)	6.29 (3.59–9.42)	6.02 (4.81–8.64)	0.8513
Neutrophils, 10^3^/μL	4.42 (2.67–7.08)	4.26 (1.62–7.75)	4.42 (2.94–7.08)	0.5732
Lymphocytes, 10^3^/μL	1.05 (0.61–1.39)	1.08 (0.71–1.46)	1.04 (0.59–1.40)	0.8187
Monocytes, 10^3^/μL	0.36 (0.26–0.51)	0.44 (0.22–0.65)	0.34 (0.29–0.48)	0.6996
Platelets, 10^3^/μL	213 (163–253.75)	196 (108.25–256.75)	215.5 (170.75–260.5)	0.3410
Creatinine, mg/dL	0.8 (0.7–1.2)	0.8 (0.7–1.2)	0.8 (0.6–1.1)	0.5497
AST, U/L	44 (29.5–70.75)	34.5 (24.25–55)	46 (31.5–86.25)	0.1429
ALT, U/L	27 (22.5–57.5)	25 (14.25–38.5)	40 (23.5–82)	0.0604
CRP, mg/L	63.55 (38.7–88.7)	47.3 (21.48–65.45)	78 (46.45–128.3)	**0.0067**
D-dimer, ng/mL	357 (179–721)	345.5 (256–775.8)	363 (178.5–786.5)	0.9510
LDH, U/L	289 (218–369)	277 (195.8–348.3)	353 (227–423)	0.1661
**Maximum oxygen therapy** [*n* (%)]				0.1756
Low-flow systems	15 (41.7)	10 (55.6)	5 (27.8)	
CPAP/NIV/OTI	21 (58.3)	8 (44.4)	13 (72.2)	
**Outcome** [*n* (%)]				>0.9999
Death	1 (2.8)	1 (5.6)	0 (0)	
Dismissal	35 (97.2)	17 (94.4)	18 (100)	

IQR, interquartile range; P_a_O_2_, arterial partial pressure of oxygen; F_i_O_2_, fraction of inspired oxygen; AST, aspartate aminotransferase; ALT, alanine aminotransferase; CRP, C reactive protein; LDH, lactate dehydrogenase; CPAP, continuous positive airway pressure; NIV, noninvasive ventilation; OTI, orotracheal intubation. ∗Statistical analyses, Mann-Whitney *U* test, Fisher exact test, Chi-square test, as appropriate.

HIV-related characteristics of PLWH are summarized in Table 2. Sixteen (88.9%) of them were on combination antiretroviral therapy (cART), while two were cART-naïve since HIV and SARS-CoV-2 infection were diagnosed concurrently. Median CD4 T cell count was 361.5 (IQR: 210.3–653.5) cells/μL, with a median CD4/CD8 ratio of 0.4 (IQR: 0.11–1.1). Plasma HIV-RNA was undetectable (<50 copies/mL) in 15 (83.3%) PLWH. Five (27.8%) had a previous AIDS diagnosis, and median CD4 T cell *nadir* was 59 (IQR: 12.5–331.3) cells/μL.

**Table 2:** HIV-related characteristic of PLWH

**Epidemiology** [*n* (%)]	
MSM	3 (16.7)
MSW/WSM	6 (33.3)
IDU	6 (33.3)
Unknown	3 (16.7)
**Immunologic parameters** [median (IQR)]	
CD4 T cell count *nadir*, cells/μL	59 (12.5–331.3)
Current CD4 T cell count, cells/μL	361.5 (210.3–653.5)
Current CD4/CD8 ratio	0.4 (0.11–1.1)
**Current CD4 T cell count** [*n* (%)]	
<200 cells/μL	4 (22.2)
200–500 cells/μL	7 (38.9)
>500 cells/μL	7 (38.9)
**Current plasma HIV-RNA** [*n* (%)]	
Undetectable (<50 copies/mL)	15 (83.3)
Detectable (50–100 copies/mL)	1 (5.6)
Detectable (>10^5^ copies/mL)	2 (11.1)
**Previous AIDS diagnosis** [*n* (%)]	5 (27.8)
**Time from HIV diagnosis, years** [median (IQR)]	18 (6.25–34.5)
**Current cART regimen** [*n* (%)]	
INSTI-based	9 (50)
PI-based	3 (16.7)
INSTI+PI-based	1 (5.6)
NNRTI-based	3 (16.7)
None (cART-naïve)	2 (11)

MSM, men who have sex with men; MSW, men who have sex with women; WSM, women who have sex with men; IDU, injective drugs use; cART, combination antiretroviral therapy; INSTI, integrase strand transfer inhibitor; PI, protease inhibitor; NNRTI, non-nucleoside reverse transcriptase inhibitor.

### Higher SARS-CoV-2 RNAemia in PLWH

Firstly, we investigated plasma SARS-CoV-2 RNAemia to estimate whether PLWH hospitalized for acute COVID-19 pneumonia experienced a greater systemic dissemination of the virus compared to the HIV-negative individuals.^54^

**Figure 1:**
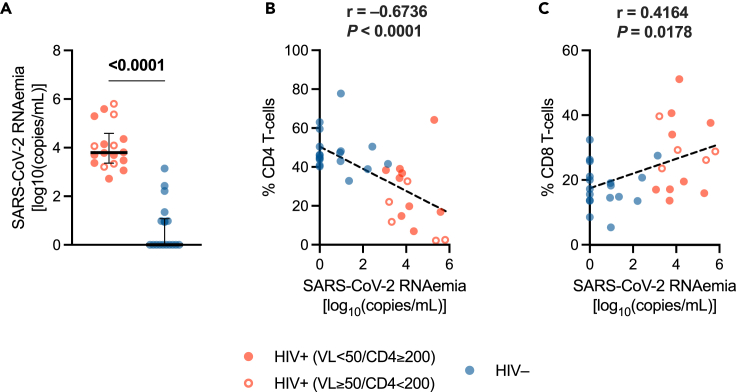
SARS-CoV-2 RNAemia and correlations with immunological parameters (A) Plasma SARS-CoV-2 RNAemia in PLWH and HIV-negative individuals. *Red/blue dots*: individual values; *bar*: median; *error bars*: interquartile range; *statistical analysis*: Mann-Whitney *U* test (*p* values < 0.05 reported above the line connecting the two groups). (B) Correlation between SARS-CoV-2 RNAemia and percentage of CD4 T cells (within lymphocytes). (C) Correlation between SARS-CoV-2 RNAemia and percentage of CD8 T cells (within lymphocytes). *Red circle*: PLWH with HIV-RNA <50 copies/mL and CD4 T cell count ≥200/μL; *open red circle*: PLWH with HIV-RNA ≥50 copies/mL and/or CD4 T cell count <200/μL; *blue circle*: HIV-negative controls; *dashed line*: simple linear regression line; *statistical analysis*: Spearman’s correlation test (r and *p* value reported above each plot). PLWH: *n* = 18; HIV-negative individuals: *n* = 18.

PLWH displayed a significantly higher RNAemia [3.796 (IQR: 3.361–4.591) log_10_ copies/mL] than HIV-negative individuals [0 (IQR: 0–1.073) log_10_ copies/mL] (*p* < 0.0001) (Figure 1A).

Of note, SARS-CoV-2 RNAemia negatively correlated with CD4 T cell percentages (r = −0.6737, *p* < 0.0001) yet positively with CD8 T cell frequencies (r = 0.4164, *p* = 0.0178) (Figures 1B and 1C). Furthermore, a negative correlation with P_a_O_2_/F_i_O_2_ *nadir* was observed (r = −0.7016, *p* < 0.001).

Taken together, these data suggest that PLWH with COVID-19 pneumonia, especially those with an imbalanced immune system and impaired respiratory function, have a limited control of SARS-CoV-2 replication/dissemination.

### Distinct plasma cytokine profile in PLWH

Next, we aimed to investigate whether PLWH also had an exacerbated cytokine storm compared to the HIV-negative individuals by measuring a panel of 12 plasma cytokines with a commercially available cytometric bead array (CBA).

**Figure 2:**
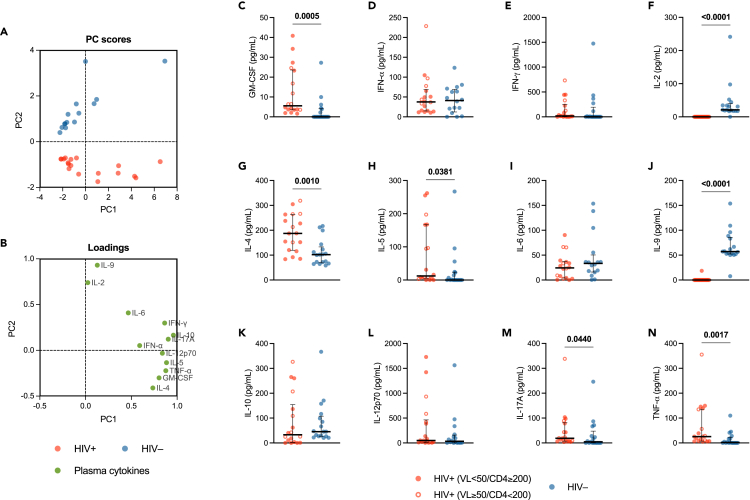
Plasma cytokines Principal component analysis (PCA) of plasma cytokines levels: (A) PC scores plot (proportion of variance for PC1: 54.13%; proportion of variance for PC2: 17.02%) showing a clear clustering of PLWH (*red dots*) and HIV-negative individuals (*blue dots*); (B) loadings plot displaying the distribution of variables (plasma cytokines) and their correlation with the principal components. Plasma concentrations (pg/mL) of GM-CSF (C), IFN-α (D), IFN-γ (E), IL-2 (F), IL-4 (G), IL-5 (H), IL-6 (I), IL-9 (J), IL-10 (K), IL-12p70 (L), IL-17A (M), and TNF-α (N) in PLWH and HIV-negative individuals. *Red circle*: PLWH with HIV-RNA <50 copies/mL and CD4 T cell count ≥200/μL; *open red circle*: PLWH with HIV-RNA ≥50 copies/mL and/or CD4 T cell count <200/μL; *blue circle*: HIV-negative controls; *bar*: median; *error bars*: interquartile range; *statistical analysis*: Mann-Whitney *U* test (*p* values < 0.05 reported above the line connecting the two groups). *Red/blue dots*: individual values; *dashed line*: simple linear regression line; *statistical analysis*: Spearman’s correlation test (r and *p* value reported above each plot), statistical significance at p < 0.05. PLWH: *n* = 18; HIV-negative individuals: *n* = 17.

To this end, we firstly performed a principal component analysis (PCA) in order to examine the influence of all the cytokines simultaneously in detecting patterns of cytokine variation between the two study groups. We thereby found a clear separation between PLWH and HIV-negative individuals based on the principal component (PC) scores (Figure 2A). The loading values assessment displayed distinct cytokines clusters (Figure 2B), suggesting that some of them had a prominent effect in determining between-group differences.

To specifically define the contribution of each cytokine, they were next analyzed singularly. Compared to HIV-negative individuals, PLWH showed higher plasma concentrations of granulocyte-macrophage colony-stimulating factor (GM-CSF) (*p* = 0.0005), IL-4 (*p* = 0.0010), IL-5 (*p* = 0.0381), IL-17A (*p* = 0.0440), and TNF-α (*p* = 0.0017) (Figures 2C, 2G, 2H, 2M, and 2N). By contrast, plasma IL-2 and IL-9 were lower in PLWH (*p* < 0.0001) (Figures 2F and 2J). Plasma IFN-α, IFN-γ, IL-6, IL-10, and IL-12p70 were detected at similar levels in the two groups (Figures 2D, 2E, 2I, 2K, and 2L).

**Figure 3:**
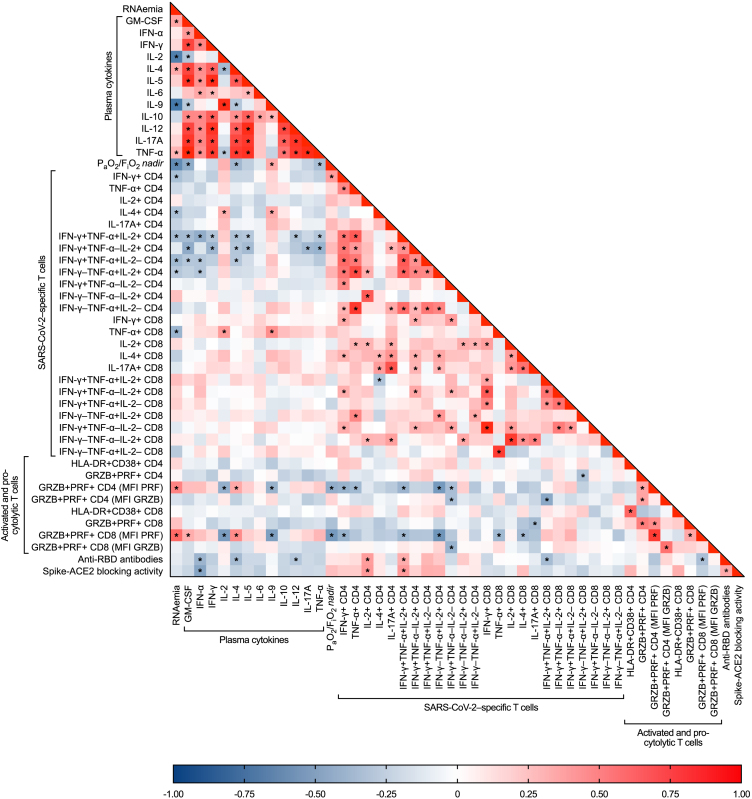
Heatmap showing correlations between SARS-CoV-2 RNAemia, plasma cytokines, clinical severity, and immune responses to SARS-CoV-2 infection *Blue cells*: negative correlations (r < 0); *red cells*: positive correlations (r > 0); *statistical analysis*: Spearman’s correlation test (∗statistical significance at p < 0.05). Study population (PLWH and HIV-negative individuals): *n* = 36. Detailed data provided in supplemental information.

SARS-CoV-2 RNAemia positively correlated with plasma GM-CSF, IL-4, and TNF-α, while negatively with IL-2 and IL-9. Likewise, P_a_O_2_/F_i_O_2_ *nadir* positively correlated with plasma GM-CSF, IL-4, and TNF-α, yet negatively with IL-9 (Figure 3; Table S1).

Altogether, these data indicate that, compared to HIV-negative individuals, PLWH with acute COVID-19 pneumonia have a distinct plasma cytokine pattern with higher levels of GM-CSF, TNF-α, IL-4, IL-5, and IL-17A, yet low circulating IL-2 and IL-9. This peculiar cytokine profile is associated with a poor control of SARS-CoV-2 replication/dissemination and a worse clinical severity in PLWH.

### Comparable T cell activation yet higher perforin production by pro-cytolytic T cells in PLWH

Having shown in PLWH a greater SARS-CoV-2 RNAemia with a distinct plasma cytokine profile, we next aimed to assess T cell activation and pro-cytolytic phenotypes via flow cytometric analysis of peripheral blood mononuclear cells (PBMCs).

**Figure 4:**
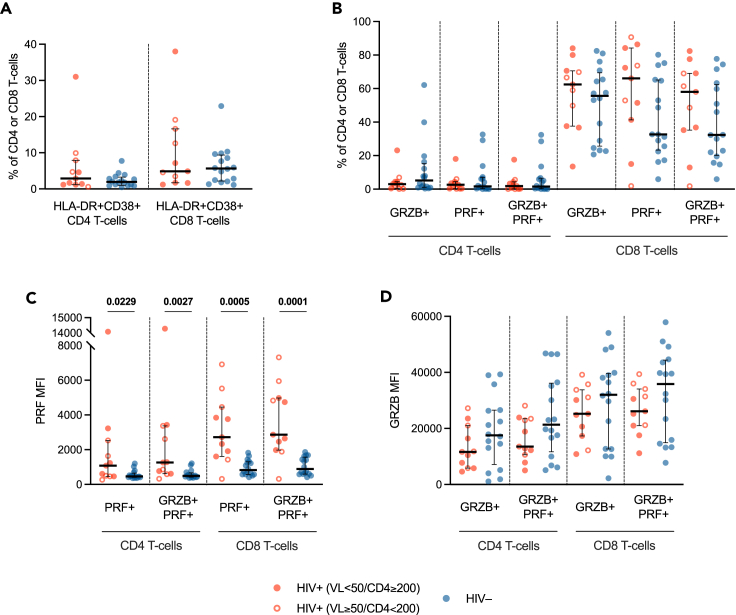
T cell activation and pro-cytolytic phenotypes (A) Frequency (percentage, %) of activated (HLA-DR+CD38+) CD4 and CD8 T cells in PLWH and HIV-negative individuals. (B) Frequency (percentage, %) of granzyme-B (GRZB)+, perforin (PRF)+, and GRZB+PRF+ CD4 and CD8 T cells. (C) Perforin production (PRF MFI) by PRF+ and GRZB+PRF+ CD4 and CD8 T cells. (D) Granzyme-B production by GRZB+ and GRZB+PRF+ CD4 and CD8 T cells. *Red circle*: PLWH with HIV-RNA <50 copies/mL and CD4 T cell count ≥200/μL; *open red circle*: PLWH with HIV-RNA ≥50 copies/mL and/or CD4 T cell count <200/μL; *blue circle*: HIV-negative controls; *bar*: median; *error bars*: interquartile range; *statistical analysis*: Mann-Whitney *U* test (*p* values < 0.05 reported above the line connecting the two groups). PLWH: *n* = 11; HIV-negative individuals: *n* = 16.

CD4 and CD8 T cell activation (as defined by coexpression of CD38 and HLA-DR) was comparable in PLWH and HIV-negative individuals (Figure 4A). Likewise, percentages of pro-cytolytic CD4 and CD8 T cells (defined as either GRZB+, PRF+, or GRZB+PRF+) were similar in the two groups (Figure 4B). However, when assessing intracellular perforin (PRF) and granzyme-B (GRZB) expression (median fluorescence intensity, MFI) by these cells, we found higher PRF production by both CD4 and CD8 pro-cytolytic T cells in PLWH (PRF+ T cells: *p* = 0.0229, *p* = 0.0005; GRZB+FRF+ T cells: *p* = 0.0027, *p* = 0.0001) (Figure 4C), despite similar GRZB expression (Figure 4D).

Interestingly, PRF production by pro-cytolytic T cells appeared to be positively correlated with SARS-CoV-2 RNAemia as well as plasma GM-CSF and IL-4, yet negatively with plasma IL-2 and IL-9 as well as P_a_O_2_/F_i_O_2_ *nadir* (Figure 3; Table S1).

These data indicate that PLWH hospitalized for SARS-CoV-2 infection do not display higher T cell activation compared to HIV-negative controls, albeit showing increased PRF production by circulating total pro-cytolytic T cells. Such elevated expression of a cytotoxic program in circulating T cells is associated with higher SARS-CoV-2 replication/dissemination, a distinct plasma cytokine *milieu* and worse respiratory function in PLWH.

### Lower magnitude and polyfunctionality of SARS-CoV-2-specific T cell responses in PLWH

To further profile the immune status of PLWH, and considered that T cell dysfunction is a hallmark of HIV infection, we next sought to assess SARS-CoV-2-specific T cells in order to determine whether the T cell compartment of the immune system adequately responded to the infection. To this end, we evaluated both quantitatively and qualitatively SARS-CoV-2-specific T cell responses by means of a flow cytometric intracellular cytokine staining (ICS) assay upon stimulation of PBMCs with a pool of 15-mer peptides (S, N, and M) of the wild-type virus.

**Figure 5:**
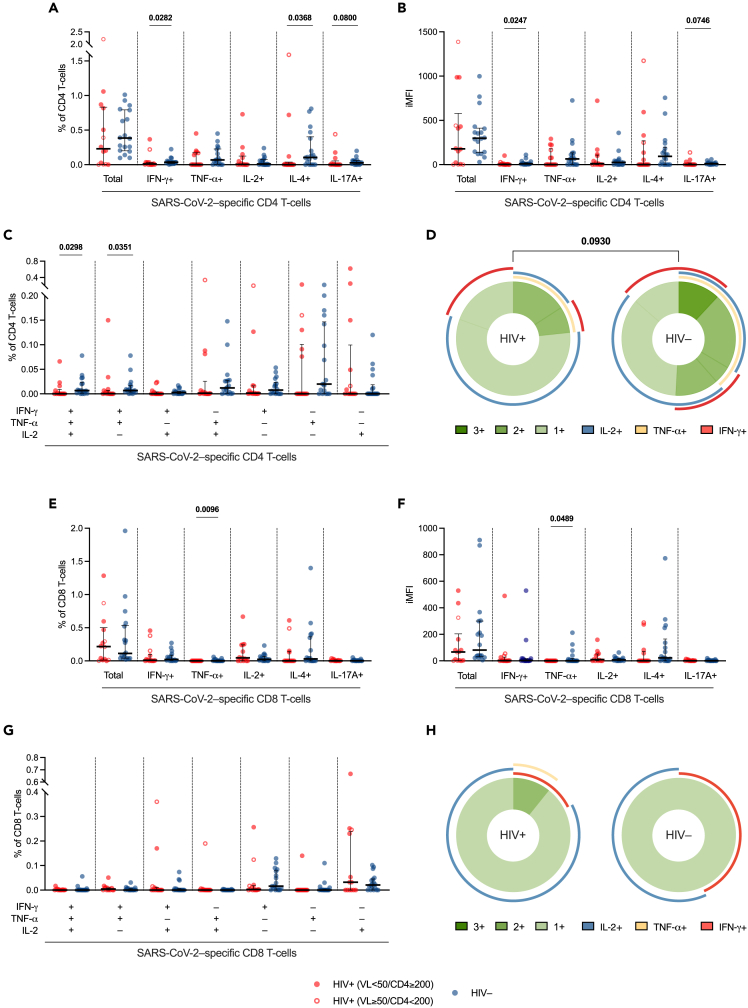
SARS-CoV-2-specific cytokine-producing CD4 and CD8 T cells Frequency (percentage, %) of SARS-CoV-2-specific cytokine (IFN-γ, TNF-α, IL-2, IL-4, IL-17A)-producing T cells within CD4 (A) and CD8 (E) T cells in PLWH and HIV-negative individuals. iMFI of SARS-CoV-2-specific cytokine (IFN-γ, TNF-α, IL-2, IL-4, IL-17A)-producing SARS-CoV-2-specific T cells within CD4 (B) and CD8 (F) T cells. Frequency of tri-, bi-, and mono-functional SARS-CoV-2-specific Th1 cytokine (IFN-γ, TNF-α, IL-2)-producing cells within CD4 (C) and CD8 (G) T cells. Donut charts showing the median distribution of polyfunctionality profiles in SARS-CoV-2-specific cytokine (IFN-γ, TNF-α, IL-2)-producing T cells within CD4 (D) and CD8 (H) T cells in PLWH and HIV-negative individuals; the donut slices represent median percentages of tri- (3+), bi- (2+), and mono- (1+) functional T cells; the arches around the circumference indicate the particular cytokine (IFN-γ, TNF-α, or IL-2) produced by the portion of T cells that lie under the arc; parts of the donut surrounded by multiple arches represent polyfunctional cells. *Red circle*: PLWH with HIV-RNA <50 copies/mL and CD4 T cell count ≥200/μL; *open red circle*: PLWH with HIV-RNA ≥50 copies/mL and/or CD4 T cell count <200/μL; *blue circle*: HIV-negative controls; *bar*: median; *error bars*: interquartile range; *statistical analyses in dot plots*: Mann-Whitney *U* test (*p* values < 0.1 reported above the line connecting the two groups); *statistical analyses between donut charts*: SPICE permutation test (*p* values < 0.1 reported above the line connecting the donut charts). SARS-CoV-2-specific T cells were measured subtracting unspecific cytokine-production in paired unstimulated control samples from stimulated samples. PLWH: *n* = 14; HIV-negative individuals: *n* = 18.

Compared to HIV-negative individuals, PLWH showed similar percentages of total cytokine-producing SARS-CoV-2-specific CD4 T cells, yet, when analyzing the intracellular production of each cytokine by these cells, we found lower percentages of SARS-CoV-2-specific CD4 T cells producing IFN-γ (*p* = 0.0282), IL-4 (*p* = 0.0368), and IL-17A (*p* = 0.0800) (Figure 5A). Accordingly, integrated median fluorescence intensity (iMFI)—a metric which incorporates both the magnitude and the quality of the immune response—for IFN-γ+ and IL-17A+ CD4 T cells was lower in PLWH (*p* = 0.0247; *p* = 0.0746) (Figure 5B). When evaluating polyfunctionality of SARS-CoV-2-specific Th1 cells, i.e., the ability to produce multiple cytokines simultaneously, we found lower IFN-γ+TNF-α+IL-2+ trifunctional, and IFN-γ+TNF-α+IL-2– bifunctional CD4 T cells in PLWH (*p* = 0.0298; *p* = 0.0351) (Figures 5C and 5D). In parallel, despite comparable total cytokine-producing SARS-CoV-2-specific CD8 T cells, PLWH showed fewer virus-specific TNF-α+ CD8 T cells, in terms of both percentage (*p* = 0.0096) and iMFI (*p* = 0.0489) (Figures 5E and 5F), yet similar CD8 polyfunctionality profiles compared to HIV-negative controls (Figures 5G and 5H).

No correlations between SARS-CoV-2-specific T cell responses and CD4 T cell counts were found in PLWH (data not shown). Remarkably, SARS-CoV-2-specific T cell responses (IFN-γ+ and IL-4+ CD4 T cells, polyfunctional Th1 cells, and TNF-α+ CD8 T cells) were negatively associated with SARS-CoV-2 RNAemia. Additionally, a positive correlation between IFN-γ+ CD4 T cells and P_a_O_2_/F_i_O_2_ *nadir* was observed. Lastly, SARS-CoV-2-specific polyfunctional Th1 cells negatively correlated with plasma cytokines, except for IL-2 and IL-9, which were positively associated with virus-specific IL-4+ CD4 and TNF-α+ CD8 T cells (Figure 3; Table S1).

Altogether, these data point to a skewed T cell response in PLWH during acute COVID-19 pneumonia, with lower magnitude and polyfunctionality of SARS-CoV-2-specific T cells. Such impaired virus-specific T cell response is linked to higher SARS-CoV-2 RNAemia, systemic inflammation, and disease severity.

### Preserved SARS-CoV-2-specific humoral responses in PLWH

Having found blunted SARS-CoV-2-specific T cell responses in PLWH, we next assessed humoral immunity by measuring anti-RBD total antibodies, by means of ELISA,^54^^,^^68^ and Spike-ACE2 binding inhibition activity, via a receptor-binding inhibition assay.^17^^,^^54^^,^^68^^,^^69^

**Figure 6:**
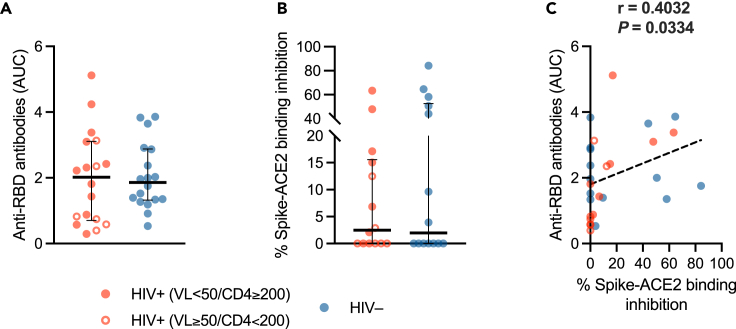
SARS-CoV-2-specific humoral immunity (A) Anti-RBD total antibodies expressed as AUC in PLWH (*n* = 18) and HIV-negative individuals (*n* = 18). (B) Spike-ACE2 binding inhibition activity expressed as percentage (%) of inhibition of ACE2-spike RBD interaction in PLWH (*n* = 14) and HIV-negative individuals (*n* = 14). *Red/blue dots*: individual values; *bar*: median; *error bars*: interquartile range; *statistical analysis*: Mann-Whitney *U* test (*p* values < 0.05 reported above the line connecting the two groups). (C) Correlation between anti-RBD antibodies and percentage of Spike-ACE2 binding inhibition. *Red circle*: PLWH with HIV-RNA <50 copies/mL and CD4 T cell count ≥200/μL; *open red circle*: PLWH with HIV-RNA ≥50 copies/mL and/or CD4 T cell count <200/μL; *blue circle*: HIV-negative controls; *dashed line*: simple linear regression line; *statistical analysis*: Spearman’s correlation test (r and *p* value reported above the plot).

Both anti-RBD antibodies and Spike-ACE2 binding inhibition activity were comparable in the two groups (Figures 6A and 6B), positively correlating with each other (r = 0.4021, *p* = 0.0334) (Figure 6C).

SARS-CoV-2-specific humoral responses did not appear to correlate with CD4 T cell counts in PLWH (data not shown). Moreover, no correlations were observed between humoral responses and SARS-CoV-2 RNAemia, plasma cytokines and P_a_O_2_/F_i_O_2_ *nadir* (Figure 3; Table S1).

These finding indicate that, despite a skewed T cell immunity, PLWH display SARS-CoV-2-specific humoral responses comparable to HIV-negative people during acute COVID-19 pneumonia.

## Discussion

In this cross-sectional study we sought to investigate SARS-CoV-2 RNAemia, plasma cytokine *milieu*, T cell activation and pro-cytolytic phenotypes, as well as SARS-CoV-2-specific T cell and humoral responses in unvaccinated PLWH hospitalized for acute COVID-19 pneumonia compared to people without HIV.

Notably, while previous research mainly focused on younger PLWH,^26^^,^^27^^,^^28^^,^^29^^,^^30^^,^^31^^,^^32^^,^^33^^,^^34^^,^^35^^,^^36^^,^^37^^,^^38^^,^^39^^,^^40^ this study included an older population with HIV, that combine aging and HIV-related features, which has been both associated with worse COVID-19 outcomes.^1^^,^^2^^,^^3^^,^^4^^,^^5^^,^^6^^,^^7^^,^^8^^,^^9^^,^^10^^,^^11^^,^^12^^,^^13^^,^^70^^,^^71^

We found that, compared to HIV-negative individuals, PLWH feature higher SARS-CoV-2 RNAemia, which is negatively correlated with CD4 T cell percentages yet positively with CD8 T cell frequencies. Since SARS-CoV-2 RNAemia has been previously shown to reflect a higher viral replication in the respiratory tract^51^ as well as dissemination of SARS-CoV-2 virions in peripheral blood,^52^ our finding suggest that PLWH may experience poor control over SARS-CoV-2 replication/dissemination, which is seemingly associated to the HIV-driven immune imbalances.^72^^,^^73^ Accordingly, as previously described in patients with immune suppression due to other causes,^74^^,^^75^^,^^76^ a delayed SARS-CoV-2 clearance in the upper respiratory tract has been proven in PLWH with severe T cell depletion.^64^^,^^67^ Likewise, in a murine model of acute SARS-CoV-2 infection, CD4 T cells depletion led to delayed viral clearance,^77^ highlighting the pivotal role of CD4 T cells in controlling viral replication. In addition, we showed that SARS-CoV-2 RNAemia appears to be negatively correlated with P_a_O_2_/F_i_O_2_ *nadir*, which is significantly lower in PLWH, pointing to a link between viral RNAemia and worse respiratory function. Such observation agrees with previous findings in the general population, as SARS-CoV-2 RNAemia has been clearly associated with severe disease and worse clinical outcomes,^52^^,^^57^^,^^58^^,^^63^ yet expands our knowledge to PLWH.

Data regarding COVID-19-related cytokine storm in PLWH are limited and controversial, variously reporting lower^33^^,^^50^ or higher^8^^,^^78^ levels of inflammatory markers. We hereby displayed that PLWH with acute COVID-19 feature a distinct plasma cytokine profile (with higher Th1/Th2/Th17-like cytokines, yet lower IL-2 and IL-9), which, in turn, is associated with poorer control of SARS-CoV-2 replication/dissemination and worse respiratory insufficiency. These data are in accordance with previous studies in HIV-negative individuals, which reported higher peripheral inflammation to be associated with both SARS-CoV-2 RNAemia^14^^,^^54^^,^^57^ and clinical severity.^14^^,^^41^^,^^44^

Immunoprofiling of total circulating T cells revealed higher intracellular PRF production by cytotoxic T cells yet similar T cell activation in PLWH compared to HIV-negative individuals. Whereas several studies have shown a hyperactivated T cell phenotype in PLWH compared to healthy controls,^79^^,^^80^^,^^81^ as well as in individuals with severe COVID-19 compared to those with mild disease,^82^^,^^83^^,^^84^^,^^85^^,^^86^^,^^87^ few data exist on the impact of HIV/SARS-CoV-2 coinfection on T cell activation, with one case report suggesting that SARS-CoV-2 infection may not further increase T cell activation in PLWH,^88^ while another study reporting higher CD38+HLA-DR+ CD4 and CD8 T cells in PLWH with SARS-CoV-2 infection.^26^ Therefore, the underlying mechanisms by which in our cohort PLWH with COVID-19 pneumonia showed similar T cell activation compared to HIV-negative controls remain to be elucidated.

We also showed that, while HIV-negative individuals develop a multi-layered SARS-CoV-2-specific immune response involving both T cell and humoral compartments at 10 days after the symptoms onset—which correspond to the temporal bridging between innate and adaptive immunity—, PLWH mount what seems to be a less coordinated adaptive immune response with Spike-binding and Spike-blocking antibodies comparable to HIV-negative controls, but lower magnitude and polyfunctionality of SARS-CoV-2-specific T cells.

Prior studies assessing adaptive immunity to SARS-CoV-2 in PLWH yielded apparently conflicting findings, which yet fit with our observations when critically considered as a whole. Two previous reports described no differences according to HIV status in SARS-CoV-2-specific T cell responses in individuals recovered from SARS-CoV-2 infection,^26^^,^^27^ albeit a positive correlation of SARS-CoV-2-specific T cell responses with CD4 T cell counts^36^ and CD4/CD8 ratio^26^ was found in PLWH. Our cohort of PLWH features lower median CD4 T cell counts and CD4/CD8 ratio than those of the aforementioned, likely explaining the skewed T cell responses to SARS-CoV-2 infection. Another study found that while virally suppressed PLWH develop similar SARS-CoV-2-specific T cell responses compared to people without HIV, those with detectable HIV viremia have significantly lower frequencies of polyfunctional antigen-specific T cells as well as impaired ability to cross-recognize viral variants.^35^ However, our cohort included only three virally unsuppressed PLWH, thus not allowing to specifically assess the contribution of uncontrolled HIV replication in hampering T cell responses against SARS-CoV-2.

Data regarding SARS-CoV-2-specific humoral immunity in PLWH are heterogeneous as well: some studies reported similar antibody responses in convalescent individuals irrespective of HIV status,^26^^,^^27^^,^^28^^,^^29^ while others—including also virally unsuppressed people—showed reduced SARS-CoV-2-specific humoral immunity in PLWH.^30^^,^^31^^,^^32^^,^^37^ However, in agreement with our data, other three recent studies proved that T cell and humoral immunity to SARS-CoV-2 do not necessarily move in the same direction, by showing that, when compared to HIV-negative individuals, convalescent PLWH developed similar humoral responses, but lower SARS-CoV-2-specific T cells.^33^^,^^34^^,^^39^ Nevertheless, the majority of aforementioned studies included individuals in the convalescent period following predominately mild disease, while our study focused on PLWH hospitalized during the acute phase of severe COVID-19, thus providing insights in such specific clinical setting.

What is more, we found that blunted SARS-CoV-2-specific T cell responses—which characterize PLWH—are associated with higher RNAemia, exacerbated cytokine storm, as well as lower P_a_O_2_/F_i_O_2_ *nadir*, while no significant correlations were found between these features and humoral responses. These data align with current evidence: while protection from infection is mediated primarily by neutralizing antibodies, protection from severe COVID-19 is mediated by a coordinated presence of antibody and T cellular immunity.^16^^,^^17^^,^^19^^,^^89^^,^^90^ Kinetic studies assessing immune responses throughout SARS-CoV-2 infection demonstrated that patients with prolonged infection and severe disease mounted robust antibody responses but had undetectable circulating SARS-CoV-2-specific T cells; on the contrary, those with a rapid expansion of IFN-γ+ SARS-CoV-2-specific T cells rapidly controlled infection without developing severe disease.^17^ Likewise, polyfunctionality of virus-specific T cells is of utmost importance, as its reduction has been shown to associate with higher SARS-CoV-2 RNAemia^54^ and worse respiratory function.^54^^,^^91^^,^^92^^,^^93^ In light of these evidences, our data suggest that impaired SARS-CoV-2 specific T cell responses in PLWH may contribute to a delayed viral clearance with possible systemic dissemination, further fueling peripheral inflammation. However, even though prior research demonstrated that a delayed engagement of antiviral immune defenses can contribute to SARS-CoV-2 dissemination via the bloodstream to distal organs,^53^ the cross-sectional nature of our study does not allow to establish causality. Therefore, we cannot rule out an alternative pathogenetic model in which a higher *ab initio* viral burden, characterized by increased SARS-CoV-2 RNAemia, might hinder the development of an effective SARS-CoV-2-specific T cell response. It is worth noting that these two models are not mutually exclusive and could potentially reinforce each other, creating a self-perpetuating cycle.

Additionally, differing from T cell activation, PRF production by pro-cytolytic T cells—which is higher in PLWH—was associated with higher SARS-CoV-2 RNAemia, higher plasma cytokines and lower P_a_O_2_/F_i_O_2_ *nadir*. These observations fit with previous literature, which reported elevated expression of a cytotoxic program in circulating T cells in SARS-CoV-2-infected patients with higher systemic inflammation^94^ and severe disease.^85^^,^^86^^,^^92^^,^^95^^,^^96^ Although we have not identified the antigenic specificity of such T cells, increases in T cells with a cytotoxic potential may result in an altered T cell landscape that could affect tissue integrity, depending on their trafficking capabilities.

Interestingly, in sharp contrast to other cytokines, we found that plasma IL-2 and IL-9 are significantly lower in PLWH. This cytokine pattern also associates with lower SARS-CoV-2-specific T cells and higher PRF production by cytotoxic T cells, as well as greater SARS-CoV-2 RNAemia and lower P_a_O_2_/F_i_O_2_ *nadir*. In keeping with these findings, reduced endogenous IL-2 production has long been acknowledged in HIV infection.^97^^,^^98^^,^^99^ Furthermore, previous studies reported higher circulating IL-2 levels as a fingerprint of asymptomatic/mild COVID-19.^100^^,^^101^ Additionally, given that IL-2 plays crucial roles in T cell survival and differentiation leading to a preferential expansion of antigen-specific clones,^102^^,^^103^^,^^104^ lower IL-2 levels may contribute to skewing SARS-CoV-2-specific T cell responses, thus leading to a less efficient control of viral replication/dissemination. Likewise, since IL-2 is a double-faced cytokine also exerting immunoregulatory activities maintaining Treg cells in a functional state,^102^^,^^103^^,^^104^ its reduced plasma concentrations might also have a role in exacerbating cytokine storm and heightening T cell cytotoxicity, likely due to perturbations in Treg cells, which has been indeed proven in severe COVID-19.^105^^,^^106^^,^^107^ By contrast, the roles of IL-9 in HIV and SARS-CoV-2 infections have not been specifically characterized to date, thus the underlying reasons for our findings concerning plasma IL-9 are unclear and remain to be elucidated.

It should also be noted that, while intracellular production of TNF-α, IL-4, and IL-17A by SARS-CoV-2-specific T cells was lower in PLWH, the plasma cytokine analysis revealed an opposite trend for the same cytokines, with lower levels in HIV-negative individuals. While such findings may appear contradictory, it must be considered that the former represent the specific antiviral response, which exert a protective effect, while the latter are expression of the cytokine storm sustained by both T lymphocytes and non-specific immune cells, which is associated with impaired viral clearance and worse respiratory insufficiency.

In conclusion, our data show that PLWH with COVID-19 pneumonia, despite preserved humoral immunity, mount skewed T cell responses with lower magnitude and polyfunctionality of SARS-CoV-2-specific T cells. This faulty virus-specific T cell response is closely intertwined with higher SARS-CoV-2 RNAemia, systemic inflammation, and disease severity. These findings suggest that additional measures to reduce the viral burden during early SARS-CoV-2 infection may be warranted in this high-risk population, and support prioritization of PLWH for SARS-CoV-2 vaccination in order to enhance T cell immunity and therefore prevent severe disease.

### Limitations of the study

Some limitations need to be acknowledged in this study. Firstly, despite clear dissimilarities in viral and immunological aspects, we did not find any statistically significant difference in mortality between the two groups, likely owing to the lack of power due to the small sample size. Secondly, study participants were enrolled during the first two waves of the COVID-19 pandemic, when the currently circulating viral variants had not yet emerged and vaccines were not available, so immune responses to the Omicron variant, which is now the most widespread all over the world, as well as the role of the vaccination and hybrid immunity, cannot be inferred. Furthermore, since the study population is primarily an older one, the findings of this study are not necessarily generalizable to a younger population. Additionally, due to the exploratory nature of this study and its small sample size, no adjustment for multiple comparison was made, so our findings need to be confirmed in larger studies. Lastly, as per cross-sectional nature of this study, all measurements were only available at a single time point during hospitalization, so that no kinetics can be derived, limiting the possibility of characterizing the dynamics of the development of immune responses in relation to SARS-CoV-2 RNAemia and systemic inflammation and, thus, establishing causality.

## STAR★Methods

### Key resources table

**Table:** 

REAGENT or RESOURCE	SOURCE	IDENTIFIER
**Antibodies**		

Viobility Fixable Dye 405/520	Miltenyi Biotec	120-028-574
CD4–APC-Vio770	Miltenyi Biotec	Cat# 130-113-223; RRID: AB_2726034
CD8–APC	Miltenyi Biotec	Cat# 130-110-679; RRID: AB_2659237
HLA-DR–VioBlue	Miltenyi Biotec	Cat# 130-111-794; RRID: AB_2652162
CD38–PE-Vio770	Miltenyi Biotec	Cat# 130-113-432; RRID: AB_2733228
Granzyme-B–PE	Miltenyi Biotec	Cat# 130-116-486; RRID: AB_2727564
Perforin–FITC	Miltenyi Biotec	Cat# 130-118-189; RRID: AB_2733686
CD8–PerCP-Vio700	Miltenyi Biotec	Cat# 130-110-682; RRID: AB_2659249
IL-17A–FITC	Miltenyi Biotec	Cat# 130-120-410; RRID: AB_2752083
IL-4–PE	Miltenyi Biotec	Cat# 130-123-698; RRID: AB_2905285
TNF-α–PE-Vio770	Miltenyi Biotec	Cat# 130-120-492; RRID: AB_2784483
IFN-γ–VioBlue	Miltenyi Biotec	Cat# 130-119-577; RRID: AB_2751736
IL-2–APC	Miltenyi Biotec	Cat# 130-111-304; RRID: AB_2652419
Goat anti-human k light chain antibody biotinylated	Bethyl Laboratories	Cat# A80-115B; RRID: AB_1966048
Goat anti-human λ light chain antibody biotinylated	Bethyl Laboratories	Cat# A80-116B; RRID: AB_1966050
Human Anti-SARS-CoV-2 Spike RBD Monoclonal Antibody, clone BIB116	Creative Diagnostics	CABT-CS044

**Biological samples**		

PBMCs	This study	N/A
Plasma	This study	N/A

**Chemicals, peptides, and recombinant proteins**		

PepTivator® SARS-CoV-2 Prot_S	Miltenyi Biotec	130-126-700
PepTivator® SARS-CoV-2 Prot_N	Miltenyi Biotec	130-126-698
PepTivator® SARS-CoV-2 Prot_M	Miltenyi Biotec	130-126-702
Phorbol myristate acetate (PMA)	Sigma-Aldrich	16561-29-8
Ionomycin	Sigma-Aldrich	56092-82-1
Brefeldin A	ThermoFisher	00-4506-51
Paraformaldehyde solution, 4% in PBS	ThermoFisher	15670799
Saponin	Sigma-Aldrich	8047-15-2
Carbonate-bicarbonate	Sigma-Aldrich	C3041-50cap
Avidin-HRP	ThermoFisher	18-4100-51
3,3’,5,5’-tetramethylbenzidine (TMB)	Invitrogen	229280010
H_2_SO_4_	Sigma-Aldrich	4803641000
Soluble human angiotensin-converting enzyme 2 (ACE2) protein fused to a human IgG1 Fc tag	InvivoGen	Fc-hace2
SARS-CoV-2 (COVID-19) S protein RBD, His Tag (MALS verified)	ACROBiosystems	SPD-C52H3
Recombinant wild-type SARS-CoV-2 RBD-HRP	Biaffin GmbH & Co KG	SPD-SR2H0-200
Lymphosep, Lymphocyte Separation Media	Biowest	L0560
Dimethyl Sulfoxide	EuroClone	67-68-5

**Critical commercial assays**		

MACSPlex Cytokine 12 kit, human	Miltenyi Biotec	130-099-169
QIAamp Viral RNA Mini Kit	QIAGEN	221413

**Oligonucleotides**		

CDC 2019-nCoV_N1 primers and probe set	Centers for Disease Control and Prevention, CDC	N/A
TaqPath™ 1-Step RT-qPCR Master Mix CG	ThermoFisher	A15299

**Software and algorithms**		

FlowLogic 8	Inivai Technologies	N/A
FlowJo 10.8	BD Biosciences	N/A
Prism 9.4	GraphPad by Dotmatics	N/A
SPICE 6.0	National Institutes of Health	N/A

**Other**		

Vacutainer**™** EDTA	BD Biosciences	367525
High-binding 96-well plates	Greiner Bio-One	655061
Fetal bovine serum	EuroClone	ECS0180L
Bovine serum albumin	Sigma-Aldrich	A7030-100G
RPMI 1640 w/o L-Glutamine	EuroClone	ECB2000
PBS Dulbecco's w/o Calcium w/o Magnesium	EuroClone	ECB4004
Penicillin-Streptomycin	Sigma	P4333-100ML
L-Glutamine	Gibco	25030-149
TWEEN® 20	Sigma	P9416-100ML
FACSVerse™	BD Biosciences	N/A
Sunrise™	TECAN	N/A
EnSight™ Multimode Plate Reader	PerkinElmer	N/A

### Resource availability

#### Lead contact

Further information and requests for resources and reagents should be directed to and will be fulfilled by the lead contact, Giulia Marchetti (giulia.marchetti@unimi.it).

#### Materials availability

This study did not generate new unique reagents.

#### Data and code availability

• All data reported in this paper will be shared by the lead contact upon request. This work is licensed under a Creative Commons Attribution 4.0 International (CC BY 4.0) license, which permits unrestricted use, distribution, and reproduction in any medium, provided the original work is properly cited. To view a copy of this license, visit https://creativecommons.org/licenses/by/4.0/.
• This paper does not report original code.
• Any additional information required to reanalyze the data reported in this paper is available from the lead contact upon request.

### Experimental model and study participant details

This study involved human participants, whose characteristics are described in Tables 1 and 2.

The study was approved by the local Ethics Committee (Comitato Etico ASST Santi Paolo e Carlo: 2020/ST/049, 2020/ST/049_BIS); written informed consent was obtained from each participant. All research was performed in accordance with the Declaration of Helsinki.

### Method details

#### Study design and population

In this cross-sectional study we consecutively enrolled unvaccinated PLWH hospitalized for ascertained SARS-CoV-2 infection (positive RT-PCR nasopharyngeal swab) and radiologically documented pneumonia at one of the participating Infectious Diseases centers in Northern Italy. Age- and sex-matched HIV-negative individuals with COVID-19 pneumonia requiring hospitalization were also enrolled as controls. Demographic and clinical characteristics of the study participants as well as HIV-related features of PLWH were collected. P_a_O_2_/F_i_O_2_ *nadir*, i.e. the ratio between arterial partial pressure of oxygen (P_a_O_2_) and fraction of inspired oxygen (F_i_O_2_) at its lowest point throughout the hospitalization, was used as a marker of the degree of respiratory insufficiency.

#### PBMCs and plasma isolation

Peripheral blood samples were collected in EDTA tubes from all study participants. Plasma was separated by centrifugation and stored at –80°C. Peripheral blood mononuclear cells (PBMCs) were obtained by Ficoll density gradient centrifugation (Lymphosep medium, Biowest), cryopreserved in fetal bovine serum (EuroClone) with 10% Dimethyl Sulfoxide (EuroClone), and stored at –80°C and then in liquid nitrogen.

#### SARS-CoV-2 RNAemia

Quantitative assessment of SARS-CoV-2 RNA was performed on frozen plasma. Briefly, viral RNA was extracted from 140 mL of plasma by using the QIAamp Viral RNA Mini Kit (QIAGEN), following the manufacturer’s instructions. 5 μL of extracted RNA was quantified by real-time PCR using the CDC 2019-nCoV_N1 primers and probe set (Centers for Disease Control and Prevention, CDC, update June 2020) and the TaqPath™ 1-Step RT-qPCR Master Mix CG (ThermoFisher). For absolute quantification, 10-fold dilutions of the 2019-nCoV_N Positive Control plasmid (Integrated DNA Technologies) were used to generate a standard curve. The assay was run in duplicate for each sample and a non-template control well was included as negative control. Quantification of the *RPP30* gene was performed to determine the quality of RNA extraction.

#### Plasma cytokines

Plasma cytokines (GM-CSF, IFN-α, IFN-γ, IL-2, IL-4, IL-5, IL-6, IL-9, IL-10, IL-12p70, IL-17A, and TNF-α) were quantified with the Human MACSPlex Cytokine 12 Kit (Miltenyi Biotec) according to the manufacturer’s instructions. Briefly, freshly thawed plasma samples were diluted 1:4 with assay diluent and incubated for 2 hours with the MACSPlex Cytokine 12 Capture Beads, followed by 1 hour of incubation with the MACSPlex Cytokine 12 Detection reagent. Samples were resuspended in 200 μL of assay buffer, acquired on a FACSVerse™ cytometer (BD Biosciences) and analyzed with FlowLogic 8 (Inivai Technologies).

#### Immunophenotyping

T cell activation and pro-cytolytic phenotype were determined by flow cytometry. Briefly, 1.5×10^6^ thawed PBMCs were plated in complete RPMI containing 10% human serum supplemented with 1% penicillin-streptomycin-glutamine. Overnight-rested PBMCs were stained with the appropriate surface antibodies for 20 minutes at 4°C in the dark; after 2% paraformaldehyde (PFA) fixation for 30 minutes at 4°C, cells were permeabilized with 0.2% saponin (Sigma-Aldrich) and stained for intracellular markers (Granzyme-B and Perforin) for 30 minutes at room temperature. Cells were then washed and resuspended in 500 μL of phosphate buffered saline (PBS). Dead cells were labeled using Viobility Fixable Dye (Miltenyi Biotec). Antibodies used were: CD4–APC-Vio770, CD8–APC, HLA-DR–VioBlue, CD38–PE-Vio770, Granzyme-B–PE and Perforin–FITC (Miltenyi Biotec).

Samples were acquired using FACSVerse™ cytometer (BD Biosciences) and FCS files were analyzed using FlowJo 10.8 (BD Biosciences). Activated T cells were defined as CD38+HLA-DR+, whereas pro-cytolytic (cytotoxic) T cells – i.e., T cells with pre-formed intracellular cytotoxic molecules – as either Granzyme-B(GRZB)+, Perforin(PRF)+, or GRZB+PRF+. GRZB and PRF were gated separately and then their coexpression was determined by using the FlowJo Boolean Gating tool (combination gates). Intracellular GRZB and PRF production by pro-cytolytic T cells was quantified by means of median fluorescence intensity (MFI). Representative plots are shown in supplemental information (Figure S1).

#### Intracellular cytokine staining (ICS) assay

SARS-CoV-2-specific T cell responses were measured by means of a flow cytometric intracellular cytokine staining (ICS) assay. Briefly, 1.5×10^6^ thawed PBMCs were plated in complete RPMI containing 10% human serum supplemented with 1% penicillin-streptomycin-glutamine. Overnight-rested PBMCs were stimulated for 5 hours with a pool of commercially available 15-mer peptides (1 μg/mL) covering the immunodominant sequence domain of the wild-type Spike (S) protein, the complete sequence of the Nucleocapsid (N) protein, and the complete sequence of the Membrane (M) protein (PepTivator SARS-CoV-2, Miltenyi Biotec). Phorbol myristate acetate (PMA) and ionomycin (Sigma-Aldrich) were used as positive control (25 ng/mL and 1 μg/mL, respectively), whereas negative controls were left untreated. Brefeldin (1 mg/mL) was added after 1 h of stimulation. Cells were harvested and stained for surface markers 20 minutes at 4°C in the dark; after 2% PFA fixation, cells were permeabilized with 0.2% saponin (Sigma-Aldrich) and stained for intracellular cytokines for 30 minutes at room temperature. Dead cells were labeled using Viobility Fixable Dye (Miltenyi Biotec). Antibodies used were: CD4–APC-Vio770, CD8–PerCP-Vio700, IL-17A–FITC, IL-4–PE, TNF-α–PE-Vio770, IFN-γ–VioBlue, IL-2–APC (Miltenyi Biotec).

Samples were acquired using FACSVerse™ cytometer (BD Biosciences) and FCS files were analyzed using FlowJo 10.8 (BD Biosciences). SARS-CoV-2-specific T cells were determined subtracting unspecific background activation in paired unstimulated samples (negative control) from stimulated samples; negative values were set to zero. SARS-CoV-2-specific T cells were expressed by percentage and integrated median fluorescence intensity (iMFI), a metric which incorporates both magnitude (frequency of cytokine-producing cells) and quality (MFI, which quantify cytokine production by such cells) of an immune response, thus reflecting the total functional response of a population of cytokine-producing T cells. T cell polyfunctionality was assessed by using the FlowJo Boolean Gating tool (combination gates) and SPICE 6.0 to identify single-, dual-, triple- cytokine-producing SARS-CoV-2-specific Th1 cells. Representative plots are shown in supplemental information (Figure S2).

#### Anti-RBD antibodies

Total anti-RBD antibodies were determined in plasma samples by a homemade ELISA. Briefly, high-binding 96-well plates (Greiner Bio-One) were coated with 3 μg/mL of recombinant wild-type SARS-CoV-2 receptor binding domain (RBD) (ACROBiosystems) diluted in 0.5 mM of carbonate-bicarbonate buffer pH 9.6 (Sigma-Aldrich) and incubated overnight at 4°C. Plates were washed with PBS-0.05%Tween-20 and blocked for 1 hour with PBS-2%BSA at 37°C. Plasma samples were serially diluted in PBS-1%BSA in triplicates (1:40, 1:240 and 1:1440), added to plates, and incubated for 2 hours at 37°C. A mix of biotinylated goat anti-human k and λ light chain were used at 1:2500 (Bethyl Laboratories, Inc., A80-115B and A80-116B) for detection, followed by avidin-HRP diluted at 1:2000 (ThermoFisher), for 30 minutes at room temperature in the dark and mild agitation. The detection was carried out with 3,3’,5,5’-tetramethylbenzidine (TMB) (Invitrogen) and quenched with 1 M H_2_SO_4_. Two plasma samples collected before the SARS-CoV-2 pandemic were included as negative controls, whereas an RBD-specific monoclonal antibody (Human Anti-SARS-CoV-2 Spike RBD Monoclonal Antibody, clone BIB116, Creative Diagnostics) was included as positive control. The optical density (OD) was measured by using TECAN Sunrise™ at 450 nm and 620 nm, and the area under the curve (AUC) was determined with GraphPad Prism 9.4.

#### Spike-ACE2 binding inhibition activity

A Spike-ACE2 inhibition assay was used to measure the ability of antibodies to block the interaction between the spike protein RBD and the ACE2 receptor, thus estimating potential viral neutralization activity, as previously described. Briefly, high-binding 96-well plates (Corning) were coated with 2 μg/mL of recombinant human ACE2-Fc (InvivoGen) diluted in 100 mM carbonate-bicarbonate buffer pH 9.6 (Sigma-Aldrich) and incubated overnight at 4°C. Plates were washed with PBS-0.05%Tween-20 and blocked with PBS-2%BSA for 1 hour at room temperature. Plasma samples were diluted 1:20 in triplicates in PBS-1%BSA and incubated with 12 ng of recombinant wild-type SARS-CoV-2 RBD-HRP (ACROBiosystems) for 1 hour at 37°C. Plates were washed and incubated with the pre-incubated plasma and RBD-HRP for 1 hour at room temperature, then detected with TMB and 1 M H_2_SO_4_. RBD-HRP alone (negative control) and plasma samples of the pre-COVID-19 era with no RBD-HRP (positive controls) were also included. The OD was measured by using EnSight (Multimode Plate Reader, PerkinElmer) at 450 nm and 570 nm. The results were expressed as percentage (%) of inhibition, calculated as [(1 – sample OD)/average negative control OD)]×100.

### Quantification and statistical analysis

Continuous variables were expressed as median (interquartile range, IQR), while categorical variables as number, *n* (percentage, %). Mann-Whitney *U* test was used for comparisons between groups for continuous variables. Fisher exact test was employed for comparison of categorical variables. Spearman’s correlation test was used for correlations between continuous variables. Principal Component Analysis (PCA) was used to visualize plasma cytokines clustering patterns in the two study groups. Data were analyzed and graphed with GraphPad Prism 9.4. Permutation test in SPICE 6.0 was employed to compare polyfunctionality patterns of SARS-CoV-2–specific Th1 cells in the two groups. *P* values less than 0.05 were considered statistically significant.

## Supplemental information

Document S1. Figures S1 and S2

Table S1. Correlation matrix (with Spearman r values, P values, and confidence intervals) related to Figure 3
