# Supplementary material for: Association between SARS-CoV-2 RNAemia, skewed T cell responses, inflammation, and severity in hospitalized COVID-19 people living with HIV
Source: iScience. 2023 Dec 7;27(1):108673. doi: 10.1016/j.isci.2023.108673 (PMC10770729; doi:10.1016/j.isci.2023.108673)
Supplement: Document S1. Figures S1 and S2 [file mmc1.pdf]

**iScience, Volume *27***

## Supplemental information

# Association between SARS-CoV-2 RNAemia, skewed T cell responses, inflammation, and severity in hospitalized COVID-19 people living with HIV

**Matteo Augello, Valeria Bono, Roberta Rovito, Camilla Tincati, Silvia Bianchi, Lucia Taramasso, Antonio Di Biagio, Annapaola Callegaro, Franco Maggiolo, Elisa Borghi, Antonella d'Arminio Monforte, and Giulia Marchetti**

# Supplemental information

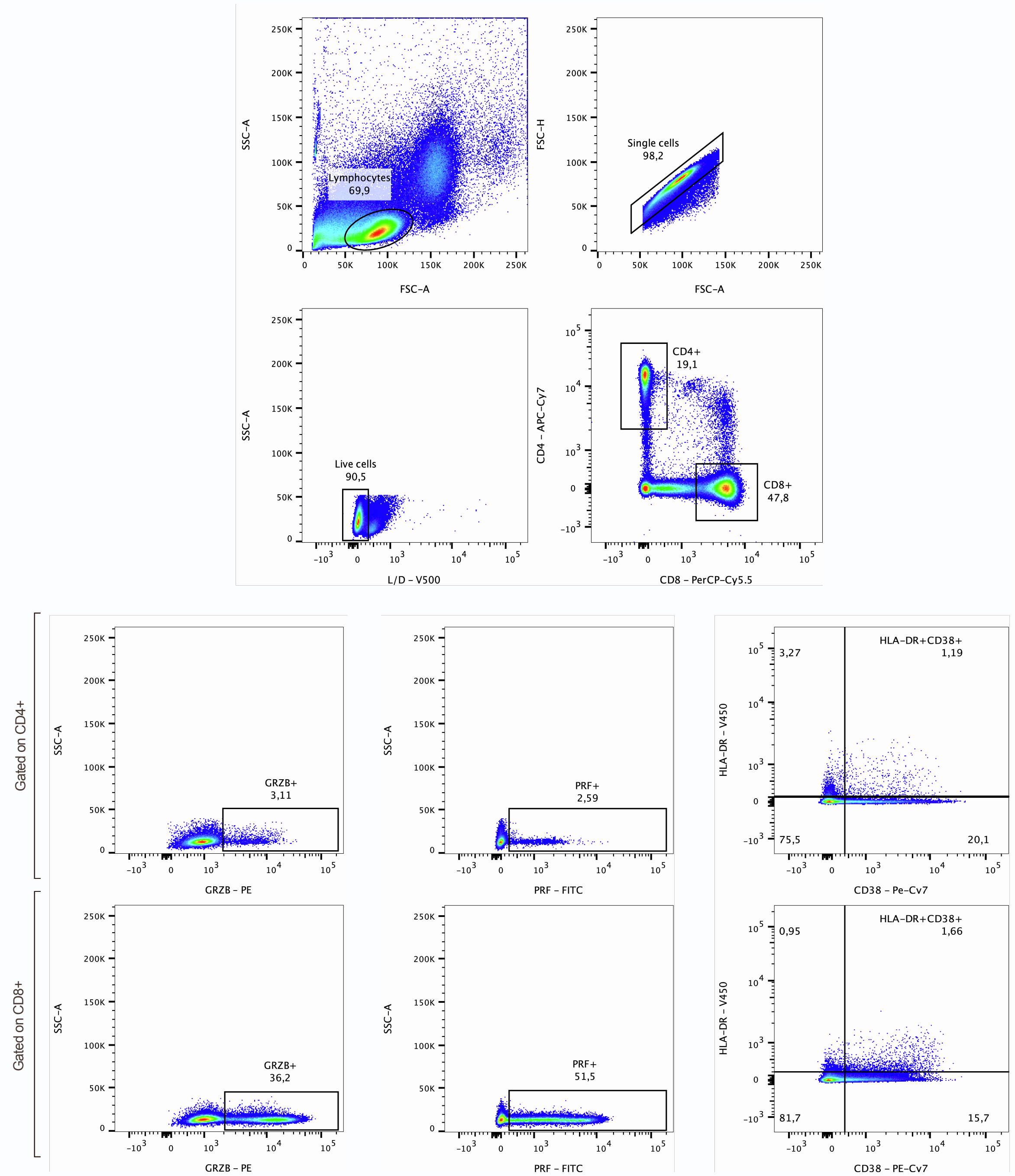


**Figure S1 (related to *Methods details, Immunophenotyping*). Representative plots of the flow cytometry gating strategy for immunophenotyping.** Lymphocytes, single and live cells were identified, and further divided into CD4+ and CD8+ cells. Granzyme-B-producing (GRZB+) and perforin-producing (PRF+) cells were determined within both CD4+ and CD8+ cells, as were activated (HLA-DR+CD38+) cells.

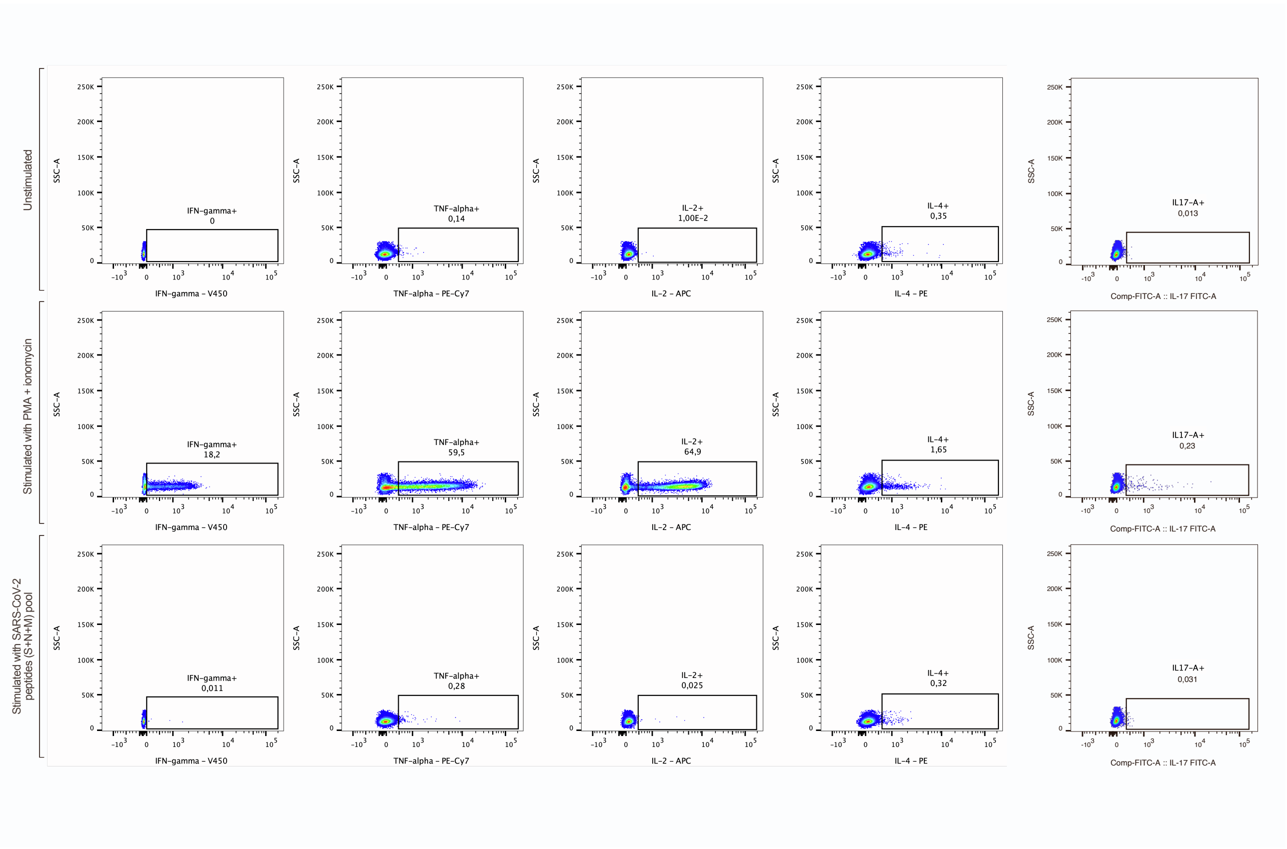


**Figure S2 (related to *Methods details, Intracellular Cytokine Staining assay*). Representative plots of the flow cytometry gating strategy for intracellular cytokine staining (ICS) assay.** Flow cytometry gating strategy used for the measurement of ICS+ (IFN-$\gamma$+, TNF-$\alpha$+, IL-2+, IL-4+, IL-17A+) CD4 and CD8 T-cells in PBMCs unstimulated, stimulated with PMA+ionomycin, and stimulated with wild-type SARS-CoV-2 S+N+M peptides pool. SARS-CoV-2–specific T-cells were measured subtracting unspecific background cytokine production in unstimulated samples from samples stimulated with SARS-CoV-2 peptides pool; samples stimulated with PMA+ionomycin were used as positive controls.
